# Latent Non-typhoidal Salmonella in Macrophages as a Potential Cause of Persistent Appendicitis

**DOI:** 10.7759/cureus.84330

**Published:** 2025-05-18

**Authors:** Shohei Takahashi, Asami Kure, Takashi Nikaido, Yoshihiro Akimoto, Kunimasa Yan

**Affiliations:** 1 Department of Pediatrics, Kosei Hospital, Tokyo, JPN; 2 Department of Pediatrics, Kyorin University Suginami Hospital, Tokyo, JPN; 3 Department of Pathology, Kosei Hospital, Tokyo, JPN; 4 Department of Anatomy, Kyorin University Faculty of Medicine, Tokyo, JPN

**Keywords:** bacterial culture, lymphoid hyperplasia, non-typhoidal salmonella infection, pediatric appendicitis, persistent infection, right lower quadrant abdominal pain, transmission electron microscopy

## Abstract

Non-typhoidal *Salmonella* (NTS) infection typically causes gastroenteritis but can sometimes lead to persistent or recurrent abdominal pain due to mesenteric lymphadenitis. Here, we present a rare case of a 10-year-old Japanese boy who experienced persistent abdominal pain caused by appendicitis in addition to mesenteric lymphadenitis due to NTS. Initially, he exhibited symptoms of gastroenteritis, including transient fever, persistent lower abdominal pain, and loose stools. Stool cultures revealed *Salmonella* O7, leading to treatment with fosfomycin. However, his abdominal pain persisted and was localized to the right lower quadrant. Physical examination suggested appendicitis, which was confirmed by ultrasound and contrast-enhanced CT findings. The patient was treated with ceftriaxone and oral cefditoren pivoxil, which relieved temporary symptoms. However, the abdominal pain recurred and worsened, necessitating an appendectomy 83 days after symptom onset.

Laparoscopic examination revealed a swollen, inflamed appendix. Bacterial culture of the appendiceal contents was positive for *Salmonella *O7. Histopathological examination showed macrophage-reactive hyperplasia in the submucosa of the lamina propria, and transmission electron microscopy identified numerous rod-shaped bacteria within macrophage lysosomes. Similar findings were observed in the mesenteric lymph nodes, indicating the persistence of *Salmonella* O7 within macrophages. The patient’s symptoms resolved rapidly following appendectomy, and stool cultures turned negative for *Salmonella* O7 one year post-infection. This case suggests that mesenteric lymph nodes may act as a long-term reservoir where macrophages harbor intracellular *Salmonella*, ultimately leading to persistent appendicitis.

Clinicians should consider appendicitis due to NTS​​​​​​​ in patients with prolonged or recurrent abdominal pain following gastroenteritis.

## Introduction

In pediatric practice, appendicitis is a common cause of abdominal pain, often necessitating emergency abdominal surgery [1]. Bacterial invasion of the gastrointestinal tract is believed to be one of the leading causes of appendicitis; however, its pathogenic mechanism and the full range of pathogenic bacteria remain unclear. A comprehensive retrospective study reviewing 579 patient records and microbiological results in pediatric appendicitis revealed many bacterial pathogens [2]. In this study, intraoperative samples were obtained either by swabbing the serosa of the appendix or by aspirating intra-abdominal fluid. The results indicated a polymicrobial spectrum in many patients, with *Escherichia* spp. detected in most cases (282/579), followed by *Bacteroides* spp. (252/579), *Streptococcus* spp. (114/579), *Pseudomonas* spp. (70/579), and *Bilophila* spp. (53/579) [2]. However, whether these bacteria act as direct pathogenic agents causing inflammation in the appendix remains unclear.

Non-typhoidal *Salmonella* (NTS) primarily causes gastrointestinal manifestations and can sometimes lead to abdominal pain mimicking appendicitis due to mesenteric lymphadenitis [3]. However, only a few case reports suggest that NTS directly causes appendicitis [4-8]. In this report, we describe a rare case of persistent abdominal pain caused by appendicitis in conjunction with mesenteric lymphadenitis due to NTS, confirmed by culture studies and histological examination, including electron microscopy.

## Case presentation

A previously healthy 10-year-old male presented to a local hospital with fever, lower abdominal pain, and diarrhea. He had no history of consuming raw food, chicken, or eggs, but had a history of caring for Japanese bantam chickens at school. His fever resolved on the fifth day, but lower abdominal pain and loose stools persisted for 12 days. Stool cultures performed at the referring hospital tested positive for *Salmonella *Braenderup, with no other significant pathogens identified. Before the culture results were known, the referring physician prescribed a seven-day course of fosfomycin. However, his abdominal pain later localized to the right lower quadrant, leading to his admission to our hospital. Physical examination revealed percussion tenderness in the right lower quadrant, but he was afebrile. Blood tests showed normal white blood cell counts, and C-reactive protein levels were undetectable (Table 1).

**Table 1:** Blood test at initial presentation CBC: complete blood count

	Investigation	Result	Reference range	
CBC	Hemogloin	14.3	14~17 g/dl	
	Hematoocrit	42.8	40~50%	
	Red blood cells	503	430~550 × 10^4^ /μｌ	
	Platelet	31.8	16~35 × 10^4^ /μｌ	
	White blood cells	8700	3300~8600 count /μｌ	
	Neutrophils	40.9	40~70%	
	Eosinophils	2.3	0~3%	
	Basophils	0.5	0~7%	
	Monocytes	8.9	3~10%	
	Lymphocytes	47.4	24~45%	
Biochemistry	Sodium	139	138~145 mmol/l	
	Potassium	4.3	3.6~4.8 mmol/l	
	Chloride	102	101~108 mmol/l	
	Blood urine nitrogen	10.2	8~20 mg/dl	
	Creatinine	0.45	0.65-~1.07 mg/dl	
	Total protein	7.8	6.6~8.1 g/dl	
	Albumin	4.6	4.1~5.1 g/dl	
	Total bilirubin	0.7	0.4~1.5 mg/dl	
	Aspartate aminotransferase	37	13~30 IU/l	
	Alanine aminotransferase	35	10~42 IU/l	
	Lactate dehydrogenase	217	124~222 IU/l	
	Glucose	86	73~109 mg/dl	
	C-reactive protein	<0.1	0~0.14 mg/dl	

Abdominal ultrasound findings indicated an enlarged appendix measuring 8 mm in diameter and an enlarged ileal lymph node measuring 15 mm in diameter. Contrast-enhanced CT revealed an enlarged appendix with a thickened wall. A stool culture obtained after admission to our hospital also yielded* Salmonella* Braenderup, which showed poor susceptibility to fosfomycin but was sensitive to third-generation cephalosporins. The patient was diagnosed with appendicitis and treated with ceftriaxone for five days, followed by oral cefditoren pivoxil for 10 days. Since his abdominal pain became mild and infrequent, he was discharged and resumed school. However, three weeks later, his right lower quadrant pain recurred and worsened. A follow-up ultrasound revealed an unchanged appendix size and persistent lymphadenopathy. The stool culture was reexamined, and *Salmonella* Braenderup was found. An appendectomy was performed 83 days after the initial onset of symptoms.

**Figure 1:**
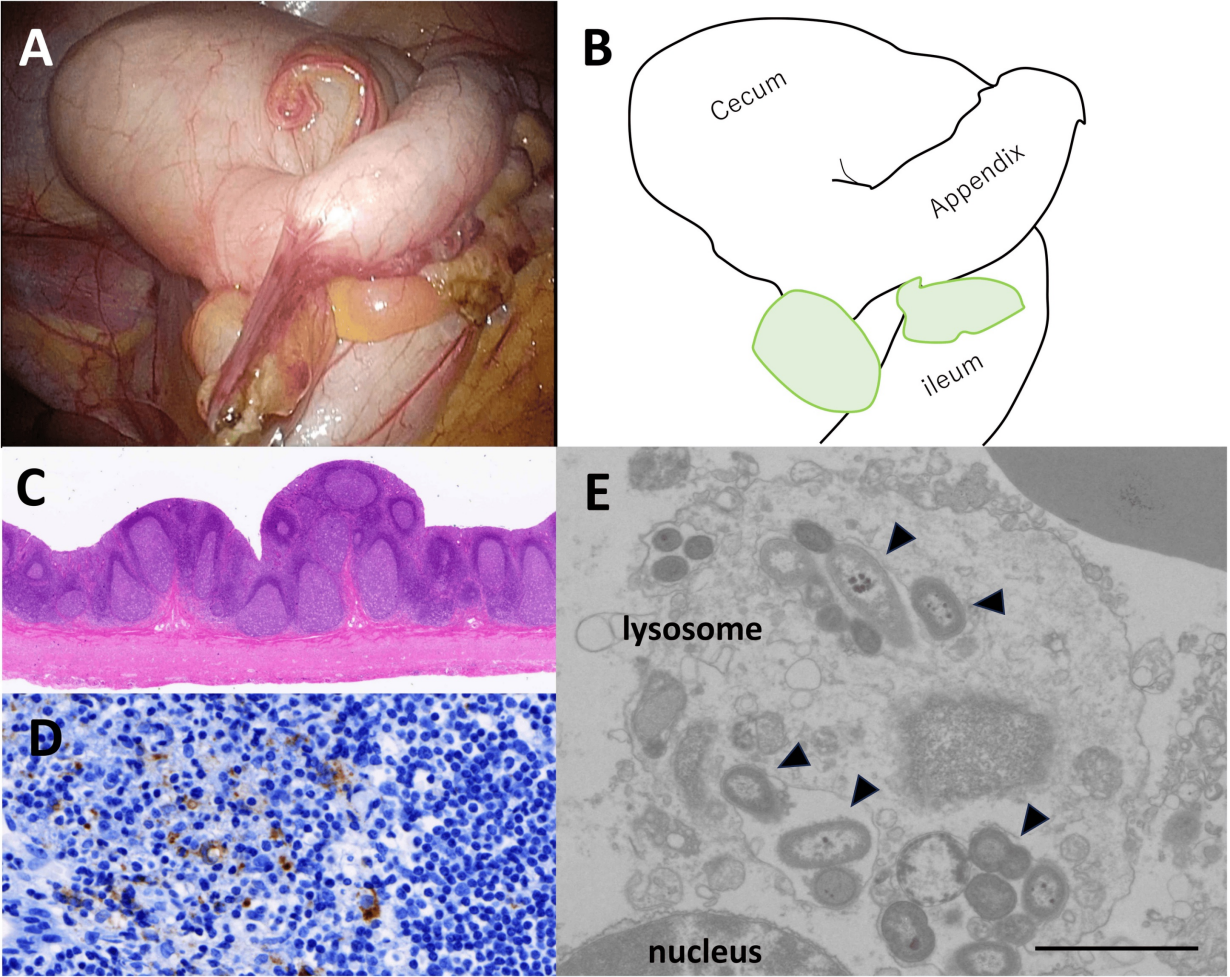
Histopathology of the appendix (A) Laparoscopy. Swollen appendix and MLN (LN1 and LN2). (B) Schematic illustration showing Figure 1A (created by Shohei Takahashi using PowerPoint). (C) Light microscopy at 100x. Hyperplastic lymphoid follicles with germinal centers in the lamina propria. (D) Immunohistochemistry of CD68 at 400x. Macrophages in the interfollicular area of the superficial mucosa (brown color). (E) Transmission electron microscopy. Multiple rod-shaped bacteria inside the lysosome of macrophages (arrowhead). Bar indicates 2 μm. MLN: mesenteric lymph node

Laparoscopic examination revealed a swollen, inflamed appendix with an associated swollen mesenteric lymph node (MLN) (Figure 1A-1B). The size of his appendix was 6 cm in the long axis. Light microscopy of hematoxylin and eosin staining revealed the preservation of the layer structure in the appendicular wall (Figure 1C). However, the lamina propria displayed prominent hyperplastic lymphoid cells with distinct germinal centers (Figure 1C). The overlying epithelium appeared atrophic and erosive (Figure 1C). Immunohistochemistry of CD68 determined obvious macrophage aggregation in the inter-follicular area of the superficial mucosa (Figure 1D). The contents of the appendix luminal side were scratched and applied to bacterial culture, which identified the presence of *Salmonella* Braenderup inside the appendix. Transmission electron microscopy was studied in the appendix to determine how *Salmonella *Braenderup existed. As shown in Figure 1E, the lysosome of macrophages was filled with numerous rod-shaped bacteria. Next, a pathology of the ileal lymph nodes was also studied (Figure 1B, LN1).

**Figure 2:**
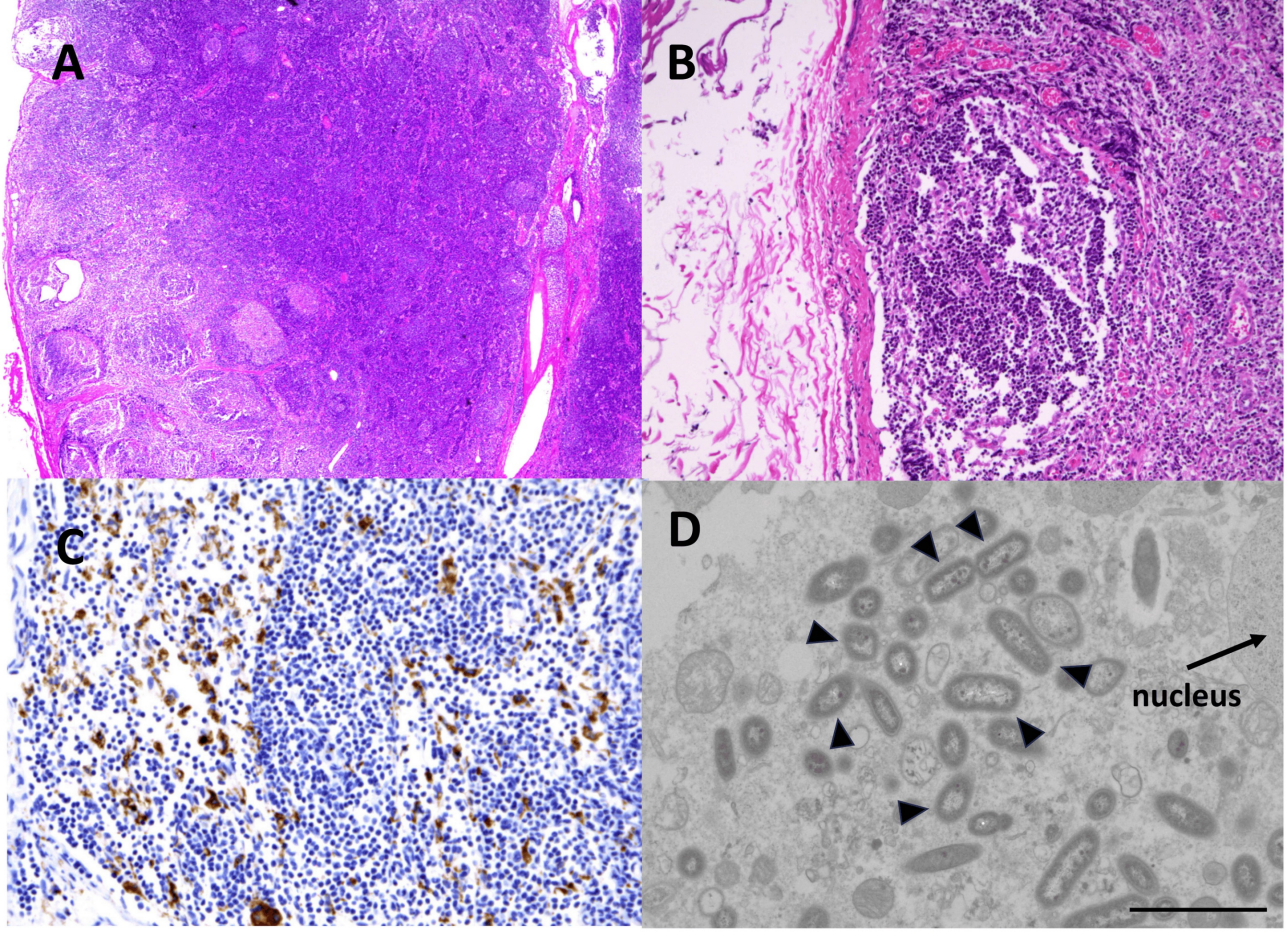
Histopathology of MLN (LN1) (A) Light microscopy with hematoxylin and eosin stain at 40x revealed follicular hyperplasia with germinal centers and interfollicular hyperplasia. (B) At 100x of (A), mild sinus histiocytosis was seen. (C) Many CD68-positive cells/macrophages were detected in the lymphatic sinus and interfollicular region (40x). (D) Transmission electron microscopy determined multiple rod-shaped bacteria (arrowhead) in the cytosol of macrophages. Bar indicates 2 μm. MLN: mesenteric lymph node

The bacterial culture of a sample scratched from the inner part of MLN indicated invasion of* Salmonella* Braenderup, as shown in an appendix. Light microscopy revealed follicular hyperplasia with germinal centers and interfollicular hyperplasia (Figure 2A), and mild sinus histiocytosis was also seen (Figure 2B). Many CD68-positive cells/macrophages were detected in the lymphatic sinus and interfollicular region (Figure 2C). Again, transmission electron microscopy determined multiple rod-shaped bacteria in the cytosol of macrophages (Figure 2D). Postoperative antibiotic therapy with cefmetazole was administered for three days. And five days after the appendectomy, his symptoms resolved completely, and he remained asymptomatic during follow-up. Stool cultures for *Salmonella* Braenderup turned negative one year after the initial infection.

## Discussion

We address two aspects of this case report: first, the clinical importance of microbiological studies in patients who experience abdominal pain that mimics appendicitis after diarrhea, and second, how NTS (*Salmonella* enteritidis) infection led to a persistent (prolonged) appendicitis. Generally, it is difficult to determine the pathogenic agents that directly cause appendicitis. Bacterial cultures from intraoperative samples may be performed, especially in complicated cases such as gangrenous and perforated appendicitis [2]. However, even such strategies do not necessarily guarantee the identification of specific pathogens directly causing inflammation of the appendix [2].

Moreover, stool culture does not help determine pathogenic agents due to the many bacteria in the intestine. In contrast, *Salmonella* species are not part of the normal intestinal flora and are typically foodborne pathogens. Globally, *Salmonella* causes approximately 78.7 million cases of gastroenteritis annually, including an estimated 59,000 deaths from NTS infections [9]. Despite the global prevalence of *Salmonella* infection, reports of appendicitis linked to either typhoidal or non-typhoidal strains remain exceedingly rare [10,11]. A few reports of *Salmonella* appendicitis may be due to the underestimation of stool cultures, which are not universally performed for appendectomy.

However, several studies have shown that delayed identification of bacteria can hinder recovery from *Salmonella*-associated appendicitis [4-7]. Our case highlights the importance of considering NTS as a potential pathogen in pediatric patients presenting with appendicitis-like symptoms following episodes of diarrhea. Timely microbiological analysis of stool and intraoperative samples is essential for selecting appropriate antibiotics, particularly in drug-resistant NTS cases, and can facilitate more rapid recovery following appendectomy.

The duration of symptomatic NTS persistence has not been clearly defined. Several studies indicated that shedding NTS in the stool ranges from 12 days to five weeks [12]. In addition, one study in Israel reported that about 2.2% of NTS patients became persistently infected, with persistence periods ranging between 30 days and 8.3 years, and that the majority (93%) of the persistently infected patients were immunocompetent [13].

Interestingly, 65% of those patients were symptomatic and developed relapsing diarrhea [13]. Overall, several factors, such as patients' age and sex, *Salmonella* serovar, and antibiotic therapy, are known to affect the clearance of *Salmonella* in the body [13]. The human body's defense mechanisms against pathogen invasion are exerted by physical barriers, innate immunity, and adaptive immunity [14]. Most NTS infections in healthy individuals do not proceed beyond the mucosa of the gastrointestinal tract, confined to the terminal ileum and colon, which results in a self-limited enterocolitis [12].

NTS breaches the epithelial barrier of the ileum portion of the small intestine either by a passive mechanism facilitated by dendritic cells or by invasion through the M cells of Peyer's patches [12]. In immunocompromised patients, invasive NTS can evade the immune system beyond this stage by entering subepithelial phagocytic cells such as macrophages, a primary cell type of innate immunity, and survive within them. Macrophages can then transport *Salmonella* via the lymphatic system and disseminate the bacteria systemically [12]. Recent findings suggest that this dissemination into extraintestinal sites occurs in immunocompromised patients and otherwise healthy humans [15].

In contrast to typhoidal *Salmonella*, the persistence sites of NTS are speculated to be other than the gallbladder because abnormalities in the biliary tract were not found to be associated with persistent NTS infections [12,13]. Indeed, the highest level of persistent Salmonella in* *the porcine host was found in ileocecal lymph nodes, jejunal lymph nodes, and tonsils, indicating that lymphatic systems are the leading persistence sites [16,17].

A murine model with *Salmonella* persistence suggested that, in the MLN, macrophages are the primary sites of *Salmonella* Typhimurium to colonize [18,19]. Macrophages are known to serve as a reservoir in which *Salmonella* can survive and replicate [20]. Human studies have not yet confirmed the presence of *Salmonella* in lymph nodes and the appendix. In this case, we first identified *Salmonella*'s actual presence in MLN and the appendix by bacterial culture using scratched samples inside both organs. Next, transmission electron microscopy identified multiple rod-shaped bacteria residing in the macrophages of MLN and the appendix. These indicate that the bacteria inside macrophages are precisely *Salmonella* Braenderup.

The existence of *Salmonella* in appendix macrophages has not been reported so far in humans; thus, this is the first report to show *Salmonella* as an intracellular parasite causing appendicitis. It is still unclear how bacteria moved into the appendix. We hypothesize that, as observed in the porcine host and murine models, the MLNs may serve as a long-term reservoir in which macrophages function as an intracellular niche, facilitating the delivery of *Salmonella* to the appendix in this case.

## Conclusions

Bacterial enterocolitis is known to cause persistent or recurrent abdominal pain during the acute diarrheal phase. However, the persistence of abdominal pain following the resolution of diarrhea warrants further diagnostic evaluation to identify potential underlying pathological conditions. In the present case, the patient exhibited persistent and recurrent abdominal pain resembling appendicitis, despite clinical resolution of NTS enterocolitis. Although a definitive causal relationship between appendicitis and NTS infection could not be established, electron microscopy, applied for the first time in humans, demonstrated the presence of NTS within macrophages in both the appendix and MLNs.

This case highlights the potential role of NTS as an etiological agent in developing persistent abdominal pain following the resolution of acute diarrheal illness. Furthermore, it emphasizes the diagnostic value of stool culture in patients presenting with appendicitis-like symptoms, particularly in cases with atypical or prolonged clinical courses.
